# Neuroprotective effects of interleukin 10 in spinal cord injury

**DOI:** 10.3389/fnmol.2023.1214294

**Published:** 2023-07-10

**Authors:** Juan Li, Pei Wang, Ting Zhou, Wenwen Jiang, Hang Wu, Shengqi Zhang, Lingxiao Deng, Hongxing Wang

**Affiliations:** ^1^Department of Rehabilitation Medicine, Zhongda Hospital Southeast University, Nanjing, China; ^2^Department of Neurological Surgery, Spinal Cord and Brain Injury Research Group, Stark Neurosciences Research Institute, Indiana University School of Medicine, Indianapolis, IN, United States

**Keywords:** IL-10, neuroprotective effects, spinal cord injury, anti-inflammatory effects, antinociceptive effects

## Abstract

Spinal cord injury (SCI) starts with a mechanical and/or bio-chemical insult, followed by a secondary phase, leading progressively to severe collapse of the nerve tissue. Compared to the peripheral nervous system, injured spinal cord is characterized by weak axonal regeneration, which leaves most patients impaired or paralyzed throughout lifetime. Therefore, confining, alleviating, or reducing the expansion of secondary injuries and promoting functional connections between rostral and caudal regions of lesion are the main goals of SCI therapy. Interleukin 10 (IL-10), as a pivotal anti-inflammatory and immunomodulatory cytokine, exerts a wide spectrum of positive effects in the treatment of SCI. The mechanisms underlying therapeutic effects mainly include anti-oxidative stress, limiting excessive inflammation, anti-apoptosis, antinociceptive effects, etc. Furthermore, IL-10 displays synergistic effects when combined with cell transplantation or neurotrophic factor, enhancing treatment outcomes. This review lists pleiotropic mechanisms underlying IL-10-mediated neuroprotection after SCI, which may offer fresh perspectives for clinical translation.

## 1. Introduction

According to the analysis from the Global Burden of Disease Study 2019, the incidence, prevalence, and years lived with disability (YLD) rate of SCI have increased worldwide (Ding et al., 2022). Globally, there were 0.9 million incident cases, 20.6 million prevalent cases and 6.2 million YLDs of total SCI in 2019. Noticeably, the incidence of SCI among older adults has sharply increased in recent decades, making the burden of disease even worse in industrialized and aging countries (Frontera and Mollett, 2017). After the pathway through which information flows, conveying locomotor, sensory, and autonomic signals, is disrupted, patients may experience dysfunctions such as difficulty standing, walking, and more. This ultimately positions SCI as the second leading cause of paralysis worldwide (Sofroniew, 2018). Although there are now many strategies trying to augment recovery following SCI, there has been no fully restorative therapy for it as yet.

SCI is artificially divided into two stages: primary and secondary injury. Initial impact causes the primary injury of spinal cord, which includes cell death, axon rupture, disruption of the blood–brain barrier, ischemia. Then, secondary injury is triggered by signaling molecules from the primary damage, such as the excitatory neurotransmitter glutamate, calcium ions, and peroxide substances. Accompanied by neutrophil infiltration, the released cytokines rapidly amplify the cascade, which induce oxidative stress, apoptosis, metabolic disturbance, and inflammation in an acute/subacute process; microenvironment imbalance, demyelination, and scar formation in the chronic phase (Anjum et al., 2020). Considering the primary injury can no longer be undone, limiting the secondary injury has become a decisive strategy and method to preserve the most structure possible, which currently offers the least loss of function.

Interleukin-10 (IL-10), first described in 1989 as cytokine synthesis inhibitory factor, controls the synthesis and releases of pro-inflammatory cytokines such as interleukin 1, tumor necrosis factor (Moore et al., 2001). As a prototypical anti-inflammatory mediator, IL-10 plays an essential role in protection from over-exuberant responses to pathogens and microbiota in autoimmunity, cancer, and homeostasis (Saraiva et al., 2020). Furthermore, IL-10 has been shown the beneficial effects in the nervous system, like autoimmune encephalomyelitis (Grace et al., 2017), depression (Yang et al., 2021), stroke (Cai et al., 2022), and SCI (Sabirzhanov et al., 2019). The deletion of the IL-10 gene worsened the recovery of limb function compared to IL-10 wild-type mice (Genovese et al., 2009). Although the organism would produce some endogenous IL-10 after SCI, this does not cover the needs of systemic and local immunoregulation for rapid termination of an excessive inflammatory storm and initiation of tissue repair and regeneration, especially reduced IL-10 expression with age (Zaaqoq et al., 2014; Zhang et al., 2015). It is worth mentioning that certain therapies for SCI, including exercise (Rodas et al., 2020), transcranial direct current stimulation (Tan et al., 2023), and transplantation of mesenchymal stem cells (Burchfield et al., 2008), has been shown to stimulate the production of IL-10. This suggests that the induction of IL-10 could potentially serve as a therapeutic mechanism for these treatments.

In this review, we mainly focus on describing and discussing results from experimental and clinical studies about IL-10-based treatment of SCI and explore its underlying mechanisms, though other cytokines also exhibit beneficial action in SCI repair, for example interleukin-4 (Francos-Quijorna et al., 2016), interferon-gamma (Sun et al., 2018). Given synergistic effects produced by IL-10 with other therapies, particular attention will be paid to combined application of cell transplantation, biomaterials in neuroprotection, and recovery after SCI, which holds therapeutic promise.

## 2. Expression of IL-10 (its receptor) and in SCI

IL-10 is a symmetric homodimer, which exerts biological functions by binding to its receptor, interleukin-10 receptor (IL-10R). Although IF-10 was found to be produced by cells of both the myeloid and lymphoid lineages including macrophages/monocytes and T cell subsets, it was discovered to function in the nervous system later, secreted by microglia, astrocytes, oligodendrocytes and neurons (Ledeboer et al., 2002; Zhou et al., 2009a; Saraiva et al., 2020). Specifically, it is produced mainly by activated astrocytes and macrophages/monocytes in the central nervous system (CNS), which modulate glia-mediated inflammatory responses via attaching to the high-affinity IL-10R by paracrine and autocrine interactions (Ledeboer et al., 2002). IL-10R is observed on microglia, astrocytes, oligodendrocytes and neurons (Molina-Holgado et al., 2001; Hulshof et al., 2002; Zhou et al., 2009a). The intracellular signaling cascades downstream of the IL-10R mainly include JAK1-TYK2-STAT3 and PI3K-Akt-mTORC pathway, which besides engaging in classical anti-inflammatory activity, regulate nonclassical organism homeostatic processes. For example, Zhou et al. (2009a) reported the IL-10R existed in spinal cord neurons and IL-10 played an anti-oxidative stress and an anti-apoptotic role by activating the JAK-STAT3 and PI3K/AKT pathways. Additionally, they also found wide distribution of the IL-10 receptor in embryonic spinal cord neurons. Combined with signaling cascades downstream of the IL-10R, IL-10 may provide not only neuroprotective but regenerative and plastic cues to neurons during development, such as regulating adult neurogenesis (Pereira et al., 2015).

**Figure 1:**
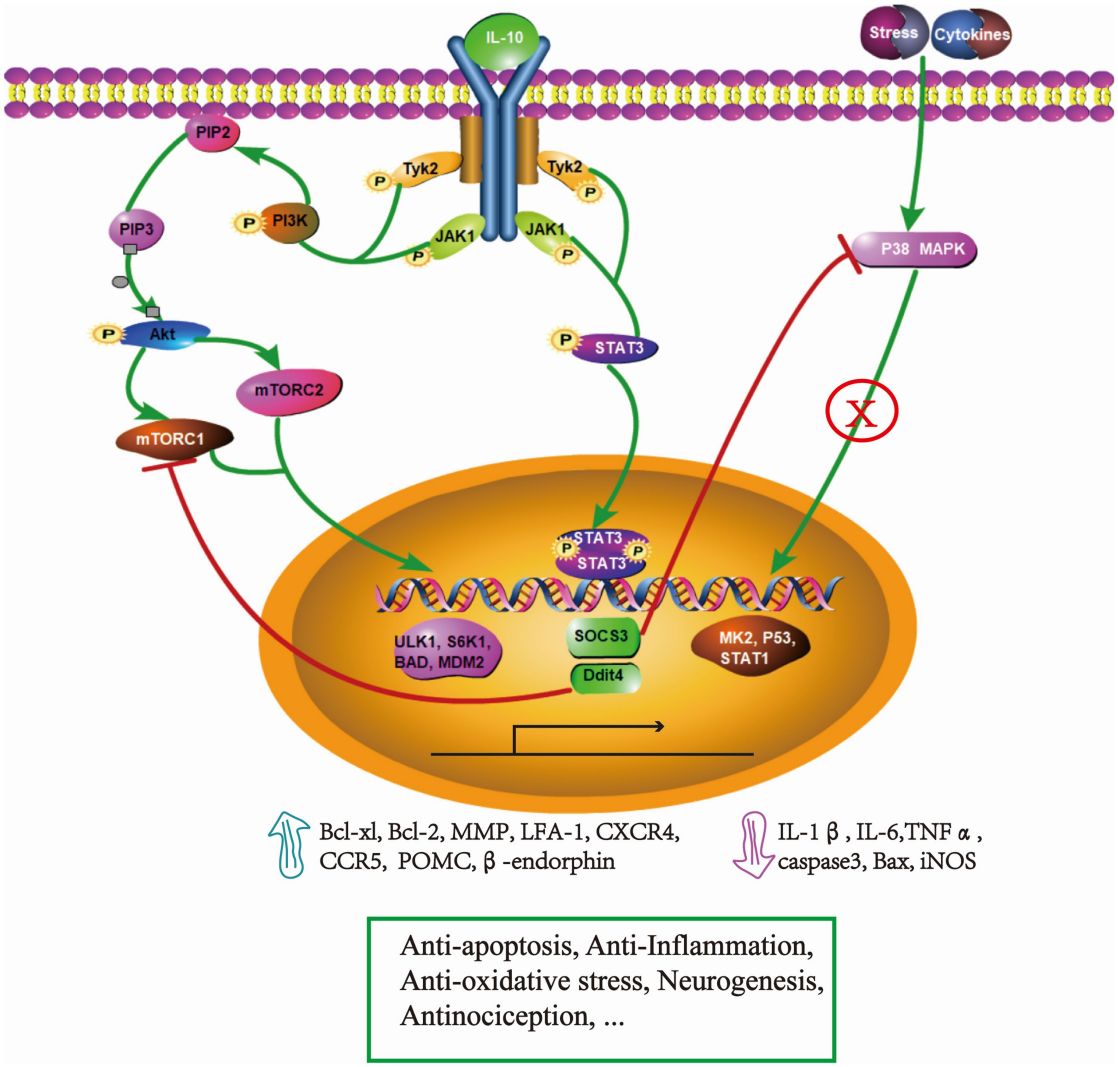
Simplified schematic representation- underlying mechanisms of IL-10 in the treatment of SCI. Binding to its receptor, IL-10 phosphorylates JAK1 and Tyk2, which activate cascaded signaling of STAT3 and PI3K/Akt/mTOR pathways. In the IL-10/STAT3 axis, the expression of SOCS3 exerts an obvious immune-inflammatory regulation and efficiently inhibits P38 MAPK pathway. This leads to a decrease of IL-1β, IL-6, TNFα, caspase 3, iNOS, which ultimately limits the secondary injury. Additionally, STAT3 activation drives the expression of β-endorphin, resulting in an antinociceptive effect. The expression of Ddit4 suppresses the activity of mTORC1 and promotes dysfunctional mitophagy, which plays an important role in the anti-oxidative stress effect. The IL-10/PI3K/Akt/mTOR pathway promotes cell survival, migration by regulating the transcription of ULK1, S6K1, BAD, MDM2 which improve the expression of anti-apoptotic protein (Bcl-xl, Bcl-2), and the surface adhesion molecules/chemokine receptors, MMP, CCR5. These events facilitate the migration of adult neural stem cells. Through these mechanisms, IL-10 demonstrates multi-neuroprotective effects in SCI. The pathway was drawn by Pathway Builder Tool 2.0.

When tissue suffers damage, IL-10 and its receptor responds quickly, which is identified by both *in vitro* and *in vivo* experiments. For example, in LPS-treated mixed glial cultures, the rise of IL-10 mRNA peaked around 1 h. Three hours after SCI, IL-10 mRNA was upregulated, however, it returned to basal levels at 7 d postinjury (Didangelos et al., 2014). Meanwhile, there is a significant increase of IL-10 protein production at 24 h after SCI, and peaked at approximately 1 or 2 weeks (Genovese et al., 2009; Mukhamedshina et al., 2017). Unfortunately, the expression of IL-10 mRNA and IL-10 protein maintained low levels in the chronic phase. Relatively, the IL-10R was found significantly labeled in motor neurons of the anterior horn after SCI, which provides structural foundation for determining whether IL-10 might have effects on neurons independent of those mediated through microglia and astrocytes (Zhou et al., 2009b). However, the more specific dynamic changes and distribution of IL-10R after SCI at different stage is still unknown. Collectively, both IL-10 and its receptor were briefly elevated in the acute phase after SCI. Nevertheless, the endogenous supply of IL-10 is not enough to repair the organism when the secondary damage storm hits. Thus, exogenous supplementation of IL-10 may suppress secondary injury and limit the damage of the SCI, which has been explored and confirmed by a great deal of studies, with the mechanisms discussed below (Figure 1).

## 3. Multi-neuroprotective effects of IL-10 in SCI

### 3.1. Anti-oxidative stress effects

In the acute phase of SCI, a spinal cord suffering mechanical or chemical damage confronts cellular necrosis, infiltration of inflammatory cells, disruption of calcium homeostasis, and glutamate-mediated excitotoxicity. Those pathological process cause mitochondrial dysfunction and production of extensive reactive oxygen/nitrogen species, which further increase the cascade reaction of secondary injury, leading to cell death (Visavadiya et al., 2016). According to an enormous amount of research, the possible mechanisms of IL-10 in the process of anti-oxidative stress is as follows. First, IL-10 enhances mitochondrial homeostasis, promotes mitophagy, and eliminates dysfunctional mitochondria characterized by low membrane potential and a high level of reactive oxygen species (Ip et al., 2017). In this research, when IL-10 signaling is absent, damaged mitochondria accumulate in macrophages which results in inflammatory storms, in turn inducing oxidative stress and reducing cellular antioxidant capacity. Furthermore, IL-10 was proved to decrease lipid peroxidation, which is the complex chain of reactions involved in oxidative damage to cellular structures and toxic processes causing cell death (Repetto et al., 2012). Vascular oxidative stress and lipid peroxidation was significantly decreased by IL-10 in an aortic remodeling model through inhibiting activation of vascular p38 and NF-κB Pathways (Qiu et al., 2022). Finally, IL-10 inhibits the production of peroxides and inflammatory cytokines, but the specific mechanism is not yet clear. For example, IL-10 treatment after SCI significantly downregulated the expression of IL1, iNOS mRNA, and iNOS protein, which effectively limited neuronal apoptosis and improved behavior function (Plunkett et al., 2001). In contrast, iNOS expression was significantly increased in spinal cord sections of IL-10 deficient mice (Genovese et al., 2009). Moreover, in a complete transection SCI mouse model, IL-10 combined with a biomaterial scaffold greatly suppressed proinflammatory cytokine production, such as iNOS, IL-1β, and TNF-α, which led to neural regeneration and axon growth (Shen et al., 2022). Interestingly, depletion of NOX2 promotes IL-10 expression following SCI, which contributes to improved functional recovery (Sabirzhanov et al., 2019). Meanwhile, Nrf2, as a chief regulator of the transcription of diverse antioxidant genes, also regulates the production of IL-10 to increases neuroprotection (Segev-Amzaleg et al., 2013). In addition to the above *in vivo* experiments, previous cell culture experiments have also showed that IL-10 counteracted proinflammatory mediators evoked by oxidative stress in Caco-2 and hepatic stellate cells through activating the mTOR-STAT3 pathway (Latorre et al., 2014; Chen et al., 2022). Collectively, oxidative stress insult plays an important role in the pathogenesis of SCI, which triggers severe and disastrous consequences including disruption of the normal cellular signaling, the breakdown of cellular structure, secretion of proinflammatory cytokines, and horribly, cell death. Therefore, restricting oxidative stress reactions will largely prevent damage from spreading, which partly can be achieved by application of IL-10.

### 3.2. Anti-inflammatory effects

Inflammation after SCI, initiates cleaning cellular debris and limiting the spread of damage. However, excessive, inappropriate inflammatory responses can exacerbate tissue injury, cause repair failure, and delayed impairment. In addition to hyper response of neutrophils, which peaked around 24 h, microglia/macrophages gradually infiltrated and peaked 7-day post-injury, followed by T-cells peaking at 9-days after injury (Hellenbrand et al., 2021). Even though microglia/macrophages decrease as the course of the disease prolongs, activated microglia/macrophages remain in the injured spinal cord for at least half a year, which may also be a potential mechanism for delayed injury (Hawthorne and Popovich, 2011). Interestingly, vast evidence shows that resisting those inflammatory cells could reduce neuroinflammation and enhance neurological function (Li et al., 2020; Poulen et al., 2021), but many studies have argued that the clearance of inflammatory cells may aggravate tissue damage (Bellver-Landete et al., 2019; Deng et al., 2022). This is due to the fact that inflammatory cells can play either a restorative or destructive role depending on specific cell subsets. It is generally accepted that M1 microglia/macrophages may exhibit harmful properties and create secondary tissue damage, whereas the M2 type may be reparative and promote tissue repair. In the acute phase of injury, neutrophils are one source of endogenous IL-10 production, but they can be suppressed by IL-10 overexpression (Sun et al., 2009). Through systemic delivery of IL-10, neutrophil proliferation and activity is reduced in SCI models; on the contrary, neutrophil activity was significantly enhanced in the SCI IL-10 deficient group (Genovese et al., 2009). The polarization of M2 microglia/macrophages is also regulated by IL-10, which means that IL-10 can even play a therapeutic role in the chronic phase. Hellenbrand et al. reported the delivery of IL-10 had significantly less “M1” cells and more “M2” cells than controls, which created a benign local microenvironment for axonal regrowth, remyelination, and functional recovery (Park et al., 2018). Shen et al. (2022) also convinced this phenomenon that IL-10-releasing hydrogel promoted the M2 macrophage/microglia phenotype, and led to neural regeneration and axon growth. Sequentially, M2 macrophages produced higher levels of IL-10 at injury spinal segments to decrease spinal cord lesion volume and resulted in increased myelination of axons and preservation of neurons, which appeared with ameliorative locomotor function (Ma et al., 2015). Lymphocytes, as executors of the immune system, maintained chronically at the injury site, which may cause trauma-induced autoimmunity after SCI (Beck et al., 2010; Jones, 2014). However, IL-10 can induce regulatory T cells, which suppressed microglia activation, decreased recruitment of peripheral monocytes, stabilized local inflammatory storm, and reduced neurodegeneration (Ishii et al., 2013; Mayo et al., 2016). While affecting inflammatory cell subtypes, IL-10 correspondingly affects the release of cytokines. IL-10 remarkably reduced TNF-a, and IL-1β production, which significantly improved functional recovery following traumatic SCI in rats (Bethea et al., 1999; Hellenbrand et al., 2019). Conversely, positive staining for TNF-α and IL-1β was significantly increased in spinal cord sections of IL-10 deficient mice (Genovese et al., 2009). In summary, the modulating-inflammatory activities of IL-10 may not only contribute to reduced secondary injury during acute and intermediate phases, but also supports neural regeneration and axon growth during the chronic stage.

### 3.3. Anti-apoptotic effects

In contrast to necrosis, a form of traumatic cell death mediated by primary injury, apoptosis signaling after SCI can be initiated by two pathways, the intrinsic pathway, which is also known as the mitochondria pathway, and the extrinsic pathway which is induced by the TNF receptor (TNFR) family (Wajant, 2002). The intrinsic pathway can result from mitochondrial dysfunction triggered by glutamate excitotoxicity, excitotoxic calcium overload, free radical-induced damage (Scholpa and Schnellmann, 2017; Slater et al., 2022), and release of harmful proinflammatory factors, such as IL-1 andTNF (Wajant, 2002). Previous reports revealed anti-apoptotic effect of IL-10 in many cells, such as those in liver (Fioravanti et al., 2017), islet (Zhu et al., 2008), nervous system (Bachis et al., 2001; Ooboshi et al., 2005). *In vitro* studies of glutamate-induced excitotoxicity revealed adult and embryonic spinal cord neurons were protected by IL-10 through Jak-Stat3 and PI3K-AKT signaling via transcription of Bcl-2 and Bcl-xL which prevented cytochrome c release and caspase 3 activation (Zhou et al., 2009a). Furthermore, another study of this research team showed that overexpression of IL-10 increased neuronal survival in the anterior quadrant of the spinal cord and improved motor function using a hemisection injury model, which correlated with increased expression of Bcl-2 and Bcl-xL in anterior quadrant neurons (Zhou et al., 2009b). In contrast, when undergoing SCI suffering, absence of IL-10 in IL-10 KO mice resulted in a significant augmentation of apoptotic cells measured by TUNEL assay, decreased Bcl-2 expression, and poorer motor function compared with IL-10 wild-type mice (Genovese et al., 2009). Given that another mechanism of apoptosis is involved in TNF-induced signaling, inhibitory effects on the production and release of TNF through IL-10 has become another anti-apoptotic pathway of IL-10 (Armstrong et al., 1996). Therefore, IL-10 may exert its anti-apoptotic effects in both direct way through Jak-Stat3 and PI3K-AKT signaling which involved in the regulation of cell cycle progression, and indirect ways by limiting release of harmful proinflammatory factors. Unnecessary apoptosis causes irreversible damage to structure and function of organism, which is one of the determining and final part of secondary injury events and also a potential target to implement therapy.

### 3.4. Anti-glial scar effects

Glial scar, mainly formed around the lesion after SCI, consists of reactive astrocytes, microglia/macrophages, and extracellular matrix molecules, especially chondroitin sulfate proteoglycan (CSPG) (Bradbury and Burnside, 2019). In the acute phase after injury, glial scarring is required for limiting the secondary injury and initiating early repair process. However, this fibrotic barrier impeded axonal regeneration and recruited immune cell, making for delayed injury in the chronic phase (Rolls et al., 2009). IL-10 was proved to attenuate astroglial reactivity by binding to its receptors on astrocytes and inhibiting the pro-inflammatory profile of activated astrocytes (Norden et al., 2014; Mayo et al., 2016) as well as inhibiting the expression of proinflammatory factors which are mediators of astroglial reactivity (Balasingam and Yong, 1996). Besides, previous reports have shown that IL-10 also can down-regulate microglial activation (Villacampa et al., 2015; Shanaki-Barvasad et al., 2022), which secretes IL-1β involving glial scar formation. Furthermore, injection of IL-10-releasing hydrogel scaffold decreased production of CSPG and suppressed formation of glial scars, with accelerated neural regeneration and axonal growth (Shen et al., 2022). Interestingly, the expression of IL-10 was promoted when glial scars were removed using ChondroitinaseABC by a p38-dependent mechanism, which offered new insight into the beneficial effects of ChABC treatment after SCI. As mentioned above, there is a dynamic equilibrium relationship between local microenvironment and glial scars, which also provides deeper insights for future SCI treatment.

### 3.5. Neurogenic and oligogliogenic effects

One of the reasons why there is no effective treatment after SCI is the limited capacity for nerve regeneration. Thus, generally, neurogenic and oligogliogenic effects of IL-10 mainly depend on neuroprotection like anti-neuronal apoptosis, as described above. However, the cell transplantation technique breaks through the limited capacity of the nervous system to regenerate; even transplanted cells face problems of survival, migration, differentiation, and functional integration in harsh microenvironments. Excitingly, overexpression of IL-10 provided a friendly microenvironment for cell transplantation therapy, which promoted 8.1% spinal progenitors’ survival (11.5-fold difference than control group) in a C5 lateral hemisection SCI model (Ciciriello et al., 2021). Yang et al. (2009) also showed that adult neural stem cells expressing IL-10 converted a hostile environment to one supportive of neurons/oligodendrocytes, which provided remyelination, and neuronal repair. This effect is even more prominent in autoimmune diseases (Klose et al., 2013). Furthermore, IL-10 also upregulates the expression of the surface adhesion molecules/chemokine receptors LFA-1, CXCR4, and CCR5, thereby enhancing adult neural stem cells migration (Guan et al., 2008). In summary, IL-10 combined with regenerative therapy may offer fresh perspectives for inflammation and immune regulation, especially at trauma-induced autoimmunity after SCI.

### 3.6. Antinociceptive effects

Neuropathic pain (NP) in SCI patients is very common, and its prevalence ranges from 18 to 96% (Li et al., 2018). This distressing and debilitating symptom results in sleep disturbances, movement disorders, and poor quality of life. Because of vague mechanisms of NP, it is very difficult to treat effectively. IL-10, as a pivotal anti-inflammatory and immunoregulatory cytokine, also exhibits antinociceptive effects in various rodent models, such as neuropathic pain (Ahmad et al., 2021), osteoarthritis (Watkins et al., 2020), and cancer pain (Apryani et al., 2020). The analgesic mechanism of IL-10, has been previously thought to inhibit the release of inflammatory factors, which have been identified as agents of pain generation. For example, IL-10 treatment resulted in a significant downregulation of IL1-β and iNOS, and limited the progression of injury-induced pain behaviors following SCI in rats (Plunkett et al., 2001). Moreover, depletion of NADPH oxidase significantly reduced mechanical/thermal cutaneous hypersensitivity and motor dysfunction after moderate contusion SCI in mice, with up-regulated expression of IL-10 (Sabirzhanov et al., 2019). Furthermore, current studies reveal that IL-10 produces antinociception in neuropathy through microglial β-endorphin expression, which is independent of the anti-inflammatory effect (Wu et al., 2018). This was confirmed by the pharmacological mechanism of gabapentin, which is recommended as a first-line treatment for neuropathic pain. Ahmad et al. (2021) illustrated that gabapentinoids alleviate NP through stimulating expression of spinal microglial IL-10 and consequent β-endorphin. Furthermore, IL-10 was also proved to reduce dorsal root ganglion neuron excitability, which is essential for controlling NP (Durante et al., 2021). In brief, NP, as an unavoidable complication in the chronic stage of spinal cord injury, needs attention. The outcome of IL-10 treatment may be unexpected and/or enticing.

## 4. Strategies to deliver IL-10 in SCI

**Figure 2:**
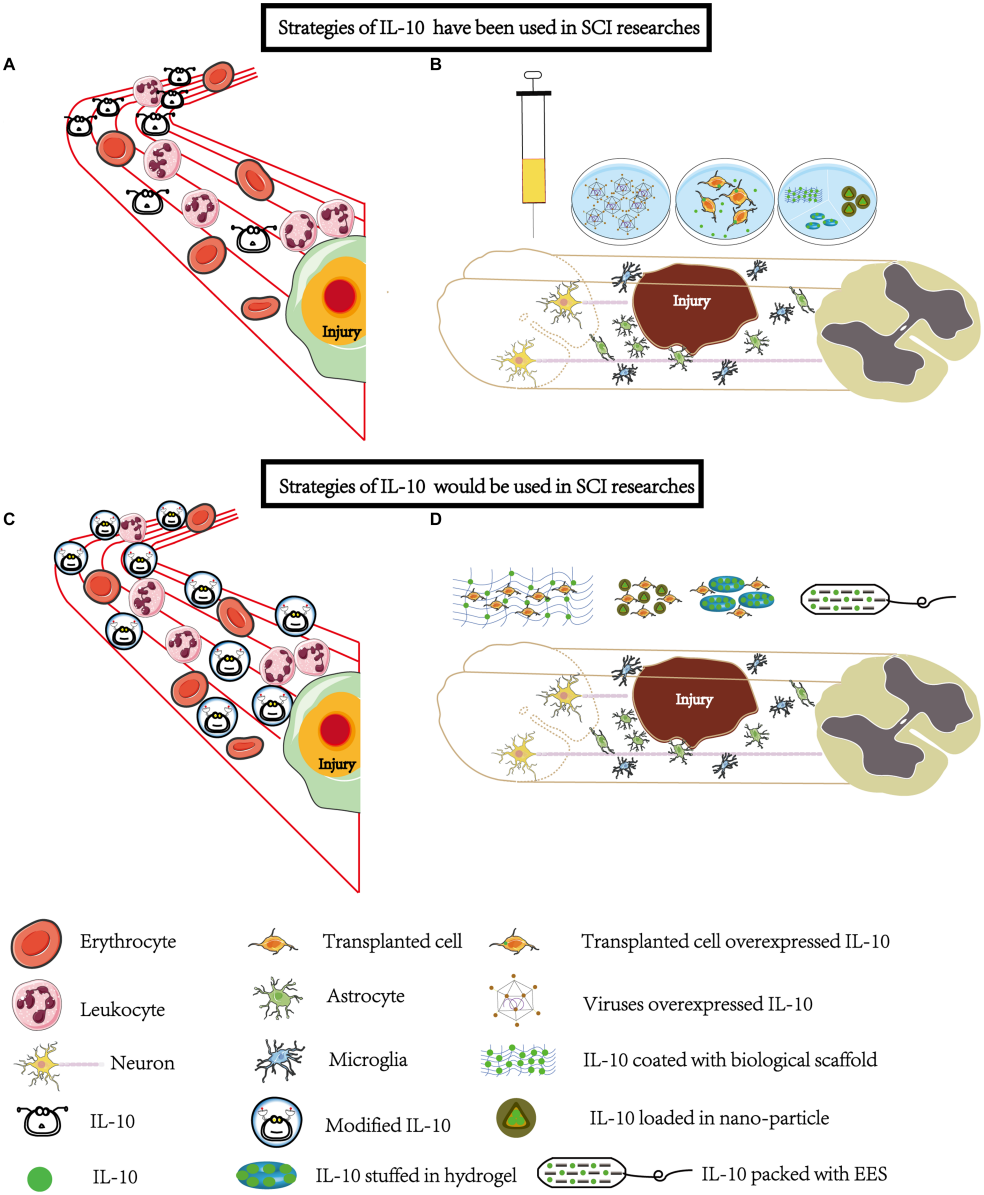
Strategies involving IL-10 have been widely explored and some more strategies will be applied in SCI research. **(A)** Systemic Administration: IL-10 has been administered systemically through intraperitoneal and intraspinal injections. However, due to its rapid metabolism, the unmodified form of IL-10 may not effectively reach the site of injury. **(B)** Local Administration: To enhance the delivery of IL-10 to the injury site, various methods have been employed. This includes using overexpressed viral vectors or combining IL-10 with biomaterials such as hydrogels, mineral-coated microparticles, nanoparticles, and scaffolds. These techniques enable localized administration and targeted release of IL-10. **(C)** Modification for Improved Stability: Modification of IL-10 can be done to improve its stability, half-life, and targeting ability. This modification allows IL-10 to focus more on the injury site. Systemic administration remains one of the most convenient methods, especially during the early stages of SCI. **(D)** Biomaterial-Based Delivery: Loading IL-10 onto biomaterials such as scaffolds, hydrogels, and nanoparticles enables a sustained and controlled release of IL-10 at the injury site. When combined with cell transplantation or epidural stimulation, IL-10 can exhibit neuroprotective and synergistic effects. It promotes the survival and migration of transplanted cells, reduces local inflammation, and may even have analgesic properties. EES, electrical epidural stimulator.

Given that the pathophysiological process of SCI is a multimolecular and multicellular interaction event, multi-mechanism-based combination strategies with IL-10 are the most desirable and efficient (Figure 2).

### 4.1. Using in a single modality approach

The earliest research on the use of IL-10 to treat SCI was more than 20 years ago. Due to the technical limitations, IL-10 was almost always administered systemically, mainly by intraperitoneal (Bethea et al., 1999; Pearse et al., 2004) and intraspinal injection (Brewer et al., 1999; Plunkett et al., 2001). In addition to being used alone (Brewer et al., 1999; Plunkett et al., 2001), it was also used in combination with other therapeutics, like methylprednisolone (Takami et al., 2002), and transplantation of Schwann cells and olfactory glia (Pearse et al., 2004). However, the results of those studies were very controversial. For example, IL-10 administered intraperitoneally reduced TNF-α production and significantly improved functional recovery following traumatic SCI in rats (Bethea et al., 1999), while another study of intraperitoneal IL-10 in the treatment of SCI showed no significant functional recovery (Takami et al., 2002). There are too many factors influencing the production of such controversial results. At first, the effective therapeutic dose for IL-10 seems to be very narrow. SCI rats receiving a single dose of IL-10 intervention exhibited a significant improvement in locomotor function two weeks after injury, whereas two doses of IL-10 failed to promote functional recovery (Bethea et al., 1999). The therapeutic window of IL-10 also matters. It significantly inhibits TNF-α production when added at 6 and 24 h after spinal cord injury, but has no effect on controlling TNF-a levels when added at 3 and 7 post-injuries (Bethea et al., 1999).

In addition, it’s worth noting that the route of administration also needs to be considered. Given that IL-10 cannot cross the blood–brain barrier (Kastin et al., 2003) and has a short half-life (Huhn et al., 1997) (2.7 to 4.5 h), single doses and cumulative doses for the treatment of SCI are huge, which can cause drug side effects and limit the clinical use of IL-10. Thereupon, with the mature application of gene editing technology, local administration of IL-10 has become the mainstream research method, such as herpes simplex virus-based vectors and poliovirus-based vectors to express IL-10 in the spinal cord in vivo (Jackson et al., 2005; Zhou et al., 2009b). Considering the enhanced efficacy of multilateral mechanisms-based treatments and extraordinary properties of biological materials, advanced exploration has focused more on those combinations, and this will be elaborated on below.

### 4.2. Combined with cell or biomaterials therapy

Novel approaches to improve the therapeutic efficacy of SCI mainly include cell transplantation-based regenerative medicine and epidural electrical stimulation (EES) in neuroprosthetic technology. Though EES has made a breakthrough progress leveraging the brain-computer interface to read cortical motor information to achieve free walking (Lorach et al., 2023), cell transplantation seems to be the promising candidates to reconstruct connections of physiological-structure. However, in addition to prove the feasibility and long-term safety of cell transplantation into the injured spinal cord in clinical trials, the clinical efficacy is vague and unreproducible changed by small sample sizes, low immune suppression, and low sensitivity study designs (Zipser et al., 2022).

As early as 1995, IL-10 was introduced as an aspect of immunosuppression to prolong graft survival (Bromberg, 1995). Excitingly, when it comes to spinal cord injury studies, overexpression of IL-10 provided a friendly microenvironment for cell transplantation therapy, which promoted 8.1% spinal progenitors’ survival (11.5-fold difference than control group) in a C5 lateral hemisection SCI model (Ciciriello et al., 2021). In another study, transplantation of IL-10-overexpressing in clinical grade mesenchymal stromal cells markedly decreased lesion volume, improved regeneration of axons, and preserved survival of neurons, accompanied with reinforced locomotor improvement in completely transected SCI, compared with naïve unmodified mesenchymal stromal cells (Gao et al., 2022). It is worth mentioning that the secretion of IL-10 by mesenchymal stem cells is considered to be one of its major therapeutic benefits in the treatment (Burchfield et al., 2008). Hence, the effect may be even better with the appropriately increased secretion of IL-10. Meanwhile, overexpression of IL-10 in mesenchymal stem cells also showed excellent neuroprotective effects and improved survival of engrafted mesenchymal stem cells in model of muscular dystrophy (Nitahara-Kasahara et al., 2021), acute ischemic stroke (Grace et al., 2017) and traumatic brain injury (Maiti et al., 2019). In the one hand, IL-10 mediated immune regulation seems to create more tolerance for those grafts (Zhang and Hill, 2019). For example, adult neural stem cells engineered to express IL-10 enhanced differentiation of transplanted cells, remyelination, and neuronal repair in experimental autoimmune encephalitis (Yang et al., 2009). In the other hand, not as a simple immunosuppressive molecule, IL-10 modulates the local microenvironment by its multipotent neuroprotective mechanisms, as already noted, to support the survival and integration with host of transplanted cell.

In addition to using vectors to overexpress IL-10 in transplanted cells, it is also designed to load into the bioengineered materials to deliver. With the advance rapidly progresses of bioengineered materials in the medical field, the sustained, stable and prolonged release of IL-10 can be achieved from the hydrogels (Ciciriello et al., 2021; Shen et al., 2022), mineral coated microparticles (Hellenbrand et al., 2019) and nanoparticles (Duncan et al., 2019). Hellenbrand et al. (2019) compared systemic administration with topical biomaterial loading of IL-10, and found that systemic IL-10 treatment attenuated TNFα and IL-1β production at 24 h post-SCI, but failed to reach significance at 7 days post-SCI, which were similar to the results of previous studies. However, IL-10 loading in the coated microparticles did remarkably attenuate the production of TNFα at 7 days post-SCI, consistent with a notable decrease in the number of M1 microglia/macrophages. IL-10 also packs with other substances into biological materials for treatment of SCI, like neurotrophins NT3 (Smith et al., 2020), as well as endogenous dangerous molecule scavenger. For example, Shen et al. (2022) developed an immunoregulatory hydrogel scaffold which can slowly release IL-10 in a complete transection SCI model. This strategy reconstructed the inflammatory balance of the immune microenvironment by combining the removal of danger signals and the addition of anti-inflammatory cytokines, leading to significantly enhanced neuroprotection and neural regeneration after SCI compared with using each of these treatments alone. Moreover, besides wrapping IL-10 proteins, recently emerged messenger RNA (mRNA)-based therapy also showed positive therapeutic effect, which sent human interleukin-10 (hIL-10)-encoding nucleoside-modified mRNA by lipid nanoparticle to the lesion cavity (Gál et al., 2023). In contrast to genetic editing that overexpresses IL-10, biomaterial loading is protected from unnecessary genetic material contamination, uncontrolled expression, and off-target problems. Nevertheless, biomaterial loading also face a series of challenges, including weak biomechanics, inappropriate degradation rate and high price (Nikolova and Chavali, 2019).

In brief, IL-10 manifests a powerful inflammation regulation and immunomodulation. Though, systemic administration of IL-10 exposes some weaknesses because of negative adjust induced by megadose, non-target and instability (Herfarth and Schölmerich, 2002; Wang et al., 2021). With application of genetic editing and biomaterials, IL-10 can be implemented locally, more targeting of the lesion and modified local environment which optimize the survival rate of endogenous and transplanted cells.

## 5. Toward to clinical applications in SCI recovery

Actually, IL-10 has been studied for more than 30 years, since it was first discovered in 1989. As early as the end of the 20th century, IL-10 entered clinical experimental research. When recombinant human IL-10 is intravenously administered in healthy volunteers, the side effects mainly consisted of mild-to-moderate flu-like symptoms which were characterized by fever with chills, headache, myalgias at the highest dose (100.0 micrograms/kg), transient decreases of lymphocyte counts, decreased platelet counts, which returned after discontinuation of the IL-10 (Huhn et al., 1996; Sosman et al., 2000). Because TNF-α and IL-1 β production were substantially and long-term inhibited during IL-10 treatment, it was previously tested in the clinical treatment of inflammatory diseases such as rheumatoid arthritis, psoriasis, and inflammatory bowel disease (Wang et al., 2019). Unfortunately, compared with placebo groups, systemic administration of IL-10 failed to result in beneficial clinical outcomes, particularly with severe dose-dependent side effect. The excellent therapeutic effect acquired from animal experiments has not been replicated in clinical patients. Despite this, Roberti et al. (2014) were the first to use low-dose cytokine therapy – Guna-Interleukin 4, Guna-Interleukin 10 and Guna-Interleukin 11- at the concentration of 10 fg/mL in psoriasis patients, and showed us the safety and efficacy of this kind of low-dose cytokine combination therapy in 2014. After that, the low-dose cytokine combination [IL-4, IL-10, and anti-IL-1 antibodies (10 fg/mL)] therapy also exhibited the promising prospect in rheumatoid arthritis, at a randomized, open, active-controlled, prospective, phase IV trial (Martin-Martin et al., 2017).

At present, there is no study of IL-10 treating SCI patients in the clinic, especially dose-dependent side effects when administered systemically, and maintenance in the preclinical stage with local delivery. The developing applications of cell and biomaterial on SCI patients occurred in recent 5 years, overcoming technical difficulties and addressing safety concerns, and achieved some positive therapeutic efficacy (Zipser et al., 2022; Lorach et al., 2023). Up to now, the first clinical study involving the transplantation of genetically modified cells into human spinal cord, is about amyotrophic lateral sclerosis at 2022, overexpressing glial cell line-derived neurotrophic factor (GDNF) (Baloh et al., 2022). Overall, it seems that there is still a long way to go before the SCI patients get benefits from application of IL-10.

Targeted delivery of IL-10 in animal models of spinal cord injury (SCI) has demonstrated significant achievements, indicating the potential application of IL-10 in clinical settings for SCI patients. However, several important considerations need to be addressed through further research. Firstly, although systemic administration of IL-10 with high doses may appear unsuitable, it remains one of the most convenient methods for treating early stages of SCI. By enhancing the structural stability and targeting of IL-10, the dosage required can be reduced. Encapsulation of IL-10 with pluronic-based nano-carriers, for example, has shown promising results with increased elimination half-life and sustained release capabilities (Kim et al., 2020). Combining IL-10 with other cytokines can also reduce the necessary dose while allowing coordinated actions with other cytokines. Additionally, utilizing biomaterials for localized delivery of IL-10 holds therapeutic promise, particularly for modulating the dysregulated local microenvironment in the intermediate-chronic phase of SCI. This approach allows for sustained, stable, and prolonged release of IL-10, enabling its pleiotropic effects (Hellenbrand et al., 2019; Shen et al., 2022). Secondly, an important consideration is determining the optimal timing for administering IL-10 treatment. Although there is no consensus on the specific duration of the treatment window, it appears that early administration of IL-10 after the injury yields greater benefits, as observed in both systemic and local administration studies (Bethea et al., 1999; Shen et al., 2022). Early administration of IL-10 has the potential to suppress an inflammatory storm, similar to the early use of tocilizumab in preventing cytokine release syndrome (CRS) (Kadauke et al., 2021). Additionally, IL-10 has the ability to regulate the phenotype of microglial cells, facilitating the shift from pro-inflammatory M1 phenotype to anti-inflammatory M2 phenotype, and inducing immunosuppression. Therefore, selecting the timing for IL-10 intervention based on the specific emphasis of the local microenvironment, such as debris clearance or tissue repair, or according to the treatment plan, is scientifically rational.

Finally, considering the complex molecular and cellular interactions involved in SCI, combining IL-10 with other therapeutic strategies is likely to lead to better treatment outcomes. Various technologies have been developed to bridge the functional communication between the areas above and below the injury site, such as cell transplantation and epidural stimulation (Harkema et al., 2011; Curtis et al., 2018; Rowald et al., 2022). When IL-10 is combined with cell transplantation technology, it has the potential to create a favorable and immunosuppressive microenvironment. Furthermore, in conjunction with epidural stimulation, IL-10 can be used in combination with stimulation devices to reduce local inflammation and provide analgesic effects, addressing the limitations and disadvantages associated with epidural stimulation techniques (Taccola et al., 2020). In conclusion, although clinical research on the use of IL-10 in SCI treatment is limited, promising results from corresponding basic research suggest its potential efficacy.

## 6. Summary

In this review, changes of IL-10 and its receptors after SCI are described. Further elucidated are the multiple-neuroprotective effects and underlying mechanisms of IL-10 in the treatment of SCI. With the application of cell and bioengineered materials, more hope has been brought to SCI patients. Since SCI is a multimolecular and multicellular interaction event which has different phases, a single approach is not a satisfactory endeavor and a meaningful combination of therapeutics is the way to go. IL-10, characterized by anti-inflammation, neuroprotection and antinociception, exhibits exceptional compatibility, which can be flexibly combined with other technologies to better achieve individualized treatment at different stages of SCI, such as anti-inflammatory in the acute phase, neuroprotective and immunosuppressive effect in association with cell transplantation at a later stage, and immunomodulatory and analgesic in the chronic stage. Although leveraging the multiple therapeutic effects of IL-10 still needs work, the research of IL-10 in SCI provides a new strategy to conquer the bottleneck of advanced treatment technology.

## Author contributions

JL: the initial draft of writing and drawing. PW and TZ: the first literature screening and collation and preliminary design. WJ, HW, and SZ: second literature screening and collation. LD and HW: critical review, commentary and revision and financial support. All authors contributed to the article and approved the submitted version.

## Funding

This work was supported by grants from the National Key Research and Development Program of China (Grant No. 2022YFC2009700), Key Medical Program of Jiangsu Commission of Health (Grant No. ZD2022048), Jiangsu Province Capability Improvement Project through Science, Technology and Education, and Jiangsu Provincial Medical Key Discipline Cultivation Unit (JSDW202202).

## Conflict of interest

The authors declare that the research was conducted in the absence of any commercial or financial relationships that could be construed as a potential conflict of interest.

## Publisher’s note

All claims expressed in this article are solely those of the authors and do not necessarily represent those of their affiliated organizations, or those of the publisher, the editors and the reviewers. Any product that may be evaluated in this article, or claim that may be made by its manufacturer, is not guaranteed or endorsed by the publisher.
